# Consensus recommendations for botulinum toxin injections in the spasticity management of children with cerebral palsy during COVID-19 outbreak

**DOI:** 10.3906/sag-2009-5

**Published:** 2021-04-30

**Authors:** Ebru YILMAZ YALÇINKAYA, Ebru YILMAZ YALÇINKAYA, Evrim KARADAĞ SAYGI, Özden ÖZYEMİŞÇİ TAŞKIRAN, Nalan ÇAPAN, Şehim KUTLAY, Birkan SONEL TUR, Özlem EL, Ece ÜNLÜ AKYÜZ, Süda TEKİN, Demet OFLUOĞLU, Murat ZİNNUROĞLU, Pınar AKPINAR, Tuğçe ÖZEKLİ MISIRLIOĞLU, Berrin HÜNER, Hakan NUR, Sibel ÇAĞLAR, Melek SEZGİN, Canan TIKIZ, Kadriye ÖNEŞ, Afitap İÇAĞASIOĞLU, Resa AYDIN

**Affiliations:** 1 Department of Physical Medicine and Rehabilitation, Gaziosmanpaşa Training and Research Hospital , University of Health Sciences, İstanbul, Turkey; 2 Department of Physical Medicine and Rehabilitation, Faculty of Medicine, Marmara University, İstanbul Turkey; 3 Department of Physical Medicine and Rehabilitation, Faculty of Medicine, Koç University, İstanbul Turkey; 4 Department of Physical Medicine and Rehabilitation, Faculty of Medicine, İstanbul University, İstanbul Turkey; 5 Department of Physical Medicine and Rehabilitation, Faculty of Medicine, Ankara University, Ankara Turkey; 6 Department of Physical Medicine and Rehabilitation, Faculty of Medicine, Dokuz Eylül University, İzmir Turkey; 7 Department of Physical Medicine and Rehabilitation, DışkapıTraining and Research Hospital, University of Health Sciences, Ankara Turkey; 8 Department Infectious Disease, Faculty of Medicine, Koç University, İstanbul Turkey; 9 Department of Physical Medicine and Rehabilitation, Faculty of Medicine, Bahçeşehir University, İstanbul Turkey; 10 Department of Physical Medicine and Rehabilitation, Faculty of Medicine, Gazi University, Ankara Turkey; 11 Department of Physical Medicine and Rehabilitation, Fatih Sultan Mehmet Training and Research Hospital, Health Science University, İstanbul Turkey; 12 Department of Physical Medicine and Rehabilitation, Faculty of Medicine, İstanbul Cerrahpaşa University, Istanbul Turkey; 13 Department of Physical Medicine and Rehabilitation, Faculty of Medicine, Akdeniz University, Antalya Turkey; 14 Department of Physical Medicine and Rehabilitation, Bakırköy Sadi KonukTraining and Research Hospital, University of Health Sciences, İstanbul Turkey; 15 Department of Physical Medicine and Rehabilitation, Faculty of Medicine, Mersin University, Mersin Turkey; 16 Department of Physical Medicine and Rehabilitation, Faculty of Medicine, Manisa Celal Bayar University, Manisa Turkey; 17 Department of Physical Medicine and Rehabilitation, İstanbul Physical Medicine and Rehabilitation Training and Research Hospital, University of Health Sciences, İstanbul Turkey; 18 Department of Physical Medicine and Rehabilitation, Faculty of Medicine, İstanbul Medeniyet University, İstanbul Turkey

**Keywords:** Botulinum toxin, cerebral palsy, COVID-19, pandemic, spasticity

## Abstract

Spasticity is the most common motor disturbance in cerebral palsy (CP). Lockdown in the COVID-19 outbreak has profoundly changed daily routines, and similarly caused the suspension of spasticity treatment plans. Besides, the delay in botulinum toxin (BoNT) injection, which is important in the management of focal spasticity, led to some problems in children. This consensus report includes BoNT injection recommendations in the management of spasticity during the COVID-19 pandemic in children with CP. In order to develop the consensus report, physical medicine and rehabilitation (PMR) specialists experienced in the field of pediatric rehabilitation and BoNT injections were invited by Pediatric Rehabilitation Association. Items were prepared and adapted to the Delphi technique by PMR specialists. Then they were asked to the physicians experienced in BoNT injections (PMR specialist, pediatric orthopedists, and pediatric neurologists) or COVID-19 (pediatric infectious disease, adult infectious disease). In conclusion, the experts agree that conservative management approaches for spasticity may be the initial steps before BoNT injections. BoNT injections can be administered to children with CP with appropriate indications and with necessary precautions during the pandemic.

## 1. Introduction

Spasticity affects up to 80% of children with cerebral palsy (CP) [1]. Management of spasticity is critical, and postponing might result in complications such as contractures, pain, skin breakdown, or functional disability [2]. On the other hand, antispasticity treatments (rehabilitation, oral medications, botulinum toxin (BoNT), intrathecal baclofen pump) require a detailed physical examination and close monitoring for appropriate treatment selection [1,3]. Close follow-up is also vital in BoNT injections, one of the first-line treatments in focal spasticity; attention must be paid to the selection of the correct muscle and the need for repeated injections within 3–6 months [4]. Lockdown in COVID-19 outbreak has profoundly changed daily routines, and similarly caused the suspension of spasticity treatment plans [3,5].

In pediatric age, the immune system is not mature, and it makes the pediatric patients susceptible to upper respiratory tract infections. Although COVID-19 is also a kind of upper respiratory tract infection at least at the beginning of the disease, the lower prevalence of COVID-19 in children is confusing [6]. Symptoms are often mild in children. They can be asymptomatic or present with most commonly fever and cough (42%). Upper respiratory tract symptoms such as nasal congestion or headache [7] or gastrointestinal symptoms such as diarrhea, vomiting, or abdominal distension can accompany [8]. Fatigue, sore throat, sputum, upper airway infections, pharyngeal erythema, tachycardia, tachypnea, pneumonia, respiratory distress, and hypoxemia are other manifestations [9].

Children with underlying diseases such as congenital heart and lung disease, chronic heart and kidney disease, hereditary metabolic, immunodeficiency diseases, malnutrition, and malignancy are likely to become severe cases for COVID-19 infection [10]. Among 48 critically ill children with COVID-19, more than 80% had major chronic diseases. Dependency on technological support, including tracheostomy, developmental delay, and seizures were other common comorbid conditions [11]. In a pediatric intensive and high-dependency care unit in France, 7 out of 27 patients had underlying neurological disease [12]. Among 130 children with confirmed COVID-19 diagnosis from 28 centers in Italy, there was one case with CP [13]. Although there is scarcely any data on whether children with CP had a higher prevalence of COVID-19, intellectual, and communication problems might complicate the interpretation of the symptoms in CP. Drooling, finger sucking, rolling over, or crawling habits might increase the possibility of viral transmission. On the other hand, mobility restrictions and dependency to parents might lead children with CP to obey the rules of “stay at home” and “social distancing” more than the able-bodied children.

Many rehabilitation centers and specialized education centers reduced their services to prevent the spread of the infection and to protect the patients and their caregivers [14]. Among 68 children receiving conventional rehabilitation for pediatric or perinatal stroke before the COVID-19 pandemic in Italy, only 5.9% continued conventional rehabilitation. Telerehabilitation and indirect remote supervision modalities were used in 23.5% and 42.6% of children, respectively. The rest did not receive any kind of rehabilitation [15]. Home programs had beneficial outcomes on the functions of the children and parent satisfaction [16]. A family-centered approach gained more importance in pediatric rehabilitation during this outbreak.

The treatment approach to CP-related spastic movement disorders should cover all conservative and surgical strategies and requires a regular interdisciplinary multimodal team approach. The success of BoNT depends on the adjunctive use of physiotherapy and splinting or casting. Delay of this effective treatment strategy that relieves pain, enhances the effects of physiotherapy, improves performance in activities of daily living, and decreases the burden of caregivers [17] caused some problems in the children during the pandemic.

On the other hand, possible systemic adverse events due to systemic diffusion of the BoNT such as generalized weakness, flu-like syndrome, fatigue, asthenia, dysphagia, and even death might complicate the symptoms of COVID-19 [18–20]. The onset of the flu-like syndrome after injection is sudden. Fever is temporary in contrast to that of COVID-19 [19]. These adverse events are more common in patients with gross motor functional classification system (GMFCS) levels IV and V []. Respiratory complication associated with anesthesia was another concern whether it suppresses the immunity against COVID-19 [18,21,22].

This consensus was planned to provide a guide especially for the physicians who apply BoNT injection to the children with CP. This report aims to answer certain questions related to BoNT injection; I. Is botulinum toxin injection safe during the COVID-19 pandemic in the pediatric population? II. Is there any difference in the selection criteria for BoNT injection during the pandemic? III. Which precautions should be taken for BoNT injection during the pandemic?

## 2. Methods

The Delphi technique was used for this consensus. It is a method used to publish recommendation or guidelines with consensus on a subject which there is not enough evidence and which the experts cannot meet simultaneously [23]. Both COGS checklist [24] and CREDES checklist, which have recommendations for Delphi methods, were reviewed and used for this study [25]. In accordance with these guidelines; as the first step, physical medicine and rehabilitation (PMR) specialists experienced in the field of pediatric rehabilitation and BoNT injections were invited by Pediatric Rehabilitation Association in Turkey, to develop a consensus report about administering BoNT injections in children with CP during the COVID-19 pandemic. Twenty physiatrists were divided into four groups of 5 physiatrists and searched the literature under four headings. These four headings are:

1) COVID-19 in children and children with disabilities,

2) Evaluation of BoNT in terms of COVID-19 risk,

3) Evaluation of BoNTinjection methods in terms of COVID-19 risk,

4) Necessary precautions during BoNT injections.

A literature search was conducted in PubMed, CINAHL, Google Scholar, Cochrane, and related online databases. Metaanalyses, systematic reviews, randomized studies, guidelines and statements, web sites of World Health Organization (WHO), and the Turkish Ministry of Health were investigated about BoNT and injection methods including anesthesia, ultrasound (US), and electromyography (EMG).The language of the literature search was English. Key words were “COVID 19 + Cerebral Palsy + Botulinum toxin”, “COVID 19 + Botulinum toxin”, “EMG + COVID 19 + Cerebral Palsy”, “Ultrasound + COVID-19”, “Anesthesia + COVID-19”,“Pediatric Anesthesia + COVID-19”,“Pulmonary + COVID-19 + Child”.

After the participants reviewed the literature and prepared items according to the patient, intervention, comparison, outcome “PICO” strategy [26], the items prepared were reviewed by four authors (NÇ, EYY, EKS, ÖÖT) separately, and relevant items were arranged in terms of language integrity and comprehensibility. A pilot test of the survey was administered to 5 physicians who did not participate in this study. Then the final version of the items adapted to the Delphi technique was approved by four authors. The final version which included 67 items was conveyed to PMR specialists (n = 34), orthopedic surgeons (n = 7), pediatric neurologists (n = 2) experienced in BoNT injection, anesthesiologists (n = 7), infectious disease specialists (n =5 ), and pediatric infectious disease specialists (n = 5) via the Qualtrics Program. For each item, the participants were asked to mark as “Agree”, “Disagree”, or “No idea/Uncertain” in the light of the existing data and their experiences. Item agreement was analyzed. Agreements more than 70% of the participants were accepted as strong, and agreements between 65% and 70% were accepted as moderate. Flow diagram of the study was shown in Figure.

**Figure:**
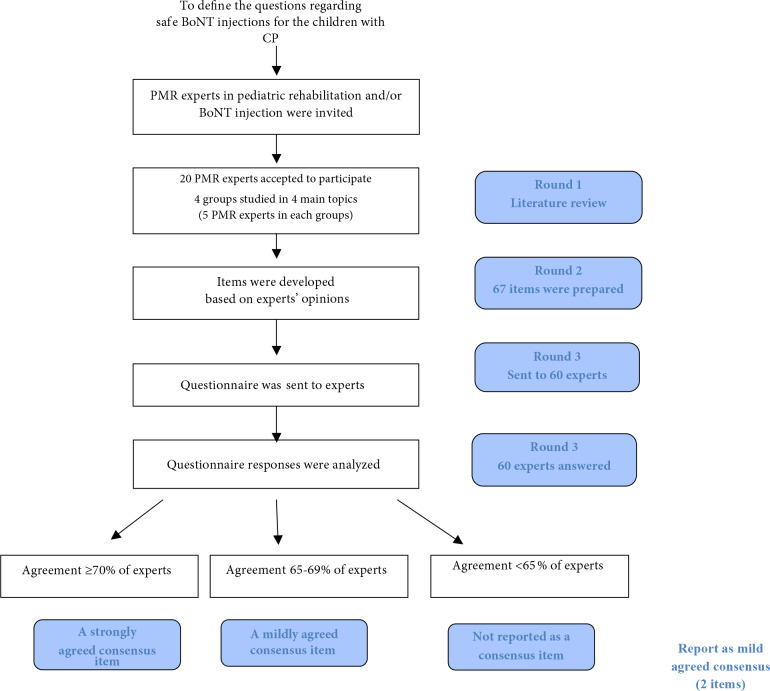
Flow diagram of the consensus and the Delphi method. (BoNT: Botulinumtoxin, CP: cerebral palsy, PMR: physical medicine and rehabilitation)

## 3. Results

Sixty experts accepted to answer Delphi questions. There were 34 PMR specialists, seven orthopedic surgeons, two pediatric neurologists, seven anesthesiologists, and five infectious and five pediatric infectious disease specialists.

In the pandemic, after lockdown was over, 13 physicians (22%) injected BoNT to the adult patients and 13 physicians (22%) to the pediatric patients. No complications were observed. In this consensus report, 25 items reached a strong agreement and were accepted as a strong recommendation, and two items had a moderate agreement.

### 3.1. Management strategies in spasticity and appropriate patient selection for BoNT injection

1. During COVID-19 pandemic, the stress and anxiety of children with disabilities and their families and closure of the rehabilitation centers may increase the spasticity of the children.

2. Rehabilitation approaches at home are effective in the development of motor functions. Other treatments instead of BoNT injections are more appropriate for children with CP including those with severe comorbidities such as dysphagia, frequent lung infections, having percutaneous endoscopic gastrostomy (PEG), or nasogastric tube. Before BoNT injection, family-oriented rehabilitation and games and telerehabilitation may be recommended.

3. BoNT injections can be administered in children with the appropriate indications during the pandemic with necessary precautions. BoNT can be postponed if there is no deterioration in functional status.

4. Telemedicine evaluation reduces the risk of COVID transmission. However, it may not be appropriate to decide for BoNT injections for every child via telemedicine alone, without face-to-face examination.

### 3.2. Evaluation before BoNT Injection

5. COVID-19 symptoms and contact history should be questioned together with other viral and bacterial infections before the injection day and should be repeated on the injection day. A separate inquiry form should also be filled for the family/caregiver.

6. Particular attention should be paid for children with intellectual and communication problems in questioning their families regarding the symptoms of their children.

7. The history of an adverse event related to previous BoNT injection should be questioned.

8. Children with GMFCS IV and V are at increased risk for more severe COVID-19 if infected.

9. Parents should be informed about the risk of COVID-19, and their consent should be obtained before the procedure.

10. Real-time reverse transcription-polymerase chain reaction (RT-PCR) test should be ordered if there is clinical suspicion of COVID-19. Children with a history of COVID-19 infection or 2-week quarantine are advised to be consulted with an infectious disease specialist. 

11. RT-PCR might be ordered before BoNT injections that will be performed in the operating room under sedation or general anesthesia (moderate agreement).

12. There is no need to repeat the RT-PCR test in a child who recovered from COVID-19 and had no symptoms left, with negative RT-PCR at least twice.

### 3.3. BoNT injection procedure

13. Going to the hospital and receiving anesthesia may increase the risk of COVID-19 infection in the children with CP.

14. Screening of body temperature at the entrance of the hospital should be performed.

15. Necessary precautions should be taken to reduce the patient’s waiting time and the number of accompanying persons.

16. For BoNT injections, sedoanalgesia or local anesthetic creams could be preferred in appropriate patients including children with severe comorbidities such as frequent lung infections or dysphagia, especially those with PEG or nasogastric tube (moderate agreement).

17. In injections under general anesthesia; complete blood count, C-reactive protein, and biochemistry might be ordered before the procedure.

18. Computed tomography (CT) should be requested only in suspected cases by consulting pediatrician/pediatric infectious disease specialists.

19. The risk of COVID-19 transmission may increase as the duration of the injection procedure gets longer. To shorten the BoNT procedure, selection of the muscles and doses should be made before entering the injection room. Electroneuromyography (EMG) and ultrasonography (US) application time should be kept as short as possible.

20. Proper disinfection of EMG cables and stimulators or the US probe and cable should be performed before and after each patient. EMG needles should be disposable.

21. Medical staff should use appropriate personal protective equipment during examination and injection. Medical staff may change their surgical masks before each patient if needed.

22. In the injection room, the number of medical staff or parent/caregiver should not be more than adequate.

23. It is not recommended to use a mask for children under 2 years old, those who have difficulty breathing, and those who cannot remove the mask without help.

24. Before the injection; body temperature, heart rate, oxygen saturation, and the frequency of breathing should be measured.

25. Hourly follow-up is sufficient for children with CP after BoNT injection. Hospitalization for monitoring after the injection may increase the risk of viral transmission. 

26. In the days following BoNT injection, the patient should be followed closely for COVID-19 symptoms.

27. Fever following BoNT injection may be confused with a symptom of COVID-19. RT-PCR test might be requested in a patient with fever and other symptoms that started 2-3 days after BoNT injection.

## 4. Discussion

This consensus report aims to define recommendations for safe BoNT injection applications both for the children with CP and healthcare workers during the pandemic. The experts agree that BoNT injections can be postponed if there is no deterioration of functional status and other antispasticity strategies may be more appropriate before BoNT injections, especially in children with severe comorbidities. There existed a strong agreement for the administration of BoNT injections to appropriate children with CP after taking necessary precautions during the pandemic. Telemedicine evaluation and telerehabilitation are options that can be benefited to reduce the risk of COVID-19 transmission. 

Before injection, COVID-19 symptoms and contact history should be carefully questioned. There is no need to order the RT-PCR test before injection routinely, and the test should be requested only in the suspected cases. The experts agreed that it is crucial to inform the parents about the risk of COVID-19 and obtain their consent before the procedure. The majority of the respondents recommended using sedoanalgesia or local anesthetic creams rather than general anesthesia during injections for eligible cases, including severe comorbidities. On the other hand, premedication may be necessary before general anesthesia to reduce airborne and droplet transmission, especially in anxious patients crying and coughing [27]. 

The current consensus suggests determining the doses and muscles before entering the injection room to shorten the duration of BoNT procedure. It is recommended not to use high doses, especially for children in GMFCS levels IV and V with higher risks for aspiration and respiratory complications [28]. Dysphagia, history of aspiration pneumonia, epilepsy, or gastrostomy has been associated with an increased risk of adverse events [29,30]. The number of medical staff should not be more than adequate similar to the literature on anesthesia procedures for pediatric patients [31]. This consensus stated that medical staff is recommended to use appropriate personal protective equipment during examination and injection and change mask for each patient if needed and proper disinfection of cable and stimulators for EMG probe and cable for the US is recommended before each patient. Patients and caregivers should wear face masks except for young children under 2 years of age, children with difficulty breathing, or those who are unable to remove the mask without help similar to Center for Disease Control and Prevention [32].

Similarly, Canadian Spasticity COVID-19 Task Force recommends cleaning all points contacted with the patients, including EMG equipment with isopropyl alcohol or a hard-surface disinfectant [33]. Some physicians prefer the portable EMG equipment instead of standard EMG machines because the disinfection of the smaller devices could be easier. The room and the neuro-diagnostic devices could be disinfected by ultraviolet-C light [34].

In another guide, US was recommended for BoNT injection during this outbreak [35]. Anesthesiologists advise wrapping US device, its probe and cable with a disposable plastic sheath [36,37]. Allam et al. suggested that the probe and the entire device should be cleaned with disinfectant wipes. The sterile disposable gel should be used for the US-guided intervention. Before each US-guided intervention, the medical staff should change N95/FFP2/KN95 or wear a surgical mask on it [35]. However, in our country, the Turkish Ministry of Health Guidelines recommend using only surgical mask in the procedures that do not produce aerosolCOVID-19 (sars-cov-2 enfeksiyonu). Çocuk hasta yönetimi ve tedavi. Bilimsel danışma kurulu çalışması [Online] (in Turkish). Web site: https://covid19.saglik.gov.tr/TR-66393/covid-19-salgin-yonetimi-ve-calisma-rehberi.html [Accessed 03 June 2020]

In the literature, there is no evidence-based data regarding the interaction of BoNT with COVID-19. In a recent paper, BoNT was proposed as a treatment against COVID-19; this hypothesis should be interpreted very carefully since it is not evidence-based [38]. More evidence is needed to infer a definitive decision. Future studies that follow the adverse events following BoNT injections during the pandemic would be valuable.

The RT-PCR test performed with nasopharyngeal and throat swabs can only be reliable in the 1st week of the disease [39]. Wang et al. reported that the sensitivity for nasal and pharyngeal swab is 63% and 32%, respectively, in the confirmed patients [40]. This consensus strongly suggested the RT-PCR test only in the clinical suspicion of COVID-19. On the other hand, there was moderate agreement to order the RT-PCR test before BoNT injections planned in the operating room under either sedation or anesthesia. Since there is no specific pathognomonic laboratory test for COVID-19 [41,42], this consensus did not recommend to order routine laboratory tests. Before general anesthesia, complete blood count and C-reactive protein are recommended. As plain chest radiography is not a specific imaging modality to diagnose or rule out COVID-19, chest CT is the recommended modality in suspected cases with consulting a pediatrician or pediatric infectious disease specialists [42].

This consensus has some limitations. COVID-19 is a novel virus and there is no definitive treatment yet. It may undergo mutation and become more or less contagious. In this case, our recommendations might need some revisions and updates. Another limitation is that there are no evidence-based studies regarding BoNT injections during the pandemic. Therefore, consensus and the Delphi method was used. Agreement over 70% was considered a strong recommendation and agreement of 65–69% as a mild recommendation. Although in some studies, 80% agreement was accepted as a strong recommendation, agreement rate was mostly reported as 70% in the literature similar to our consensus report [25].

In conclusion, BoNT injections can be administered in children with CP without COVID-19 symptoms or contact history following appropriate personal protective equipment and disinfection rules.

## References

[ref1] (2020). Medical updates in management of hypertonia. Physical Medicine and Rehabilitation Clinics of North America.

[ref2] (2020). Practical guidance for outpatient spasticity management during the coronavirus (COVID-19) pandemic: canadian spasticity COVID-19 task force. Canadian Journal of Neurological Sciences.

[ref3] (2020). Spasticity treatments during COVID-19 pandemic: clinical recommendations. Frontiers in Neurology.

[ref4] (2019). Botulinum toxin type A in the treatment of lower limb spasticity in children with cerebral palsy. Cochrane Database Systematic Reviews.

[ref5] (2020). Bloem BR. Journal of Parkinsons Disease.

[ref6] (2020). Clinical and CT features in pediatric patients with COVID-19 infection: Different points from adults. Pediatric Pulmonology.

[ref7] (2020). Diagnosis and treatment recommendations for pediatric respiratory infection caused by the 2019 novel coronavirus. World Journal of Pediatrics.

[ref8] (2020). Updated diagnosis, treatment and prevention of covid-19 in children: experts’ consensus statement (condensed version of the second edition. World Journal of Pediatrics.

[ref9] (2020). Clinical manifestations of children with COVID-19: a systematic review. PediatricPulmonology.

[ref10] (2020). -19 epidemic: disease characteristics in children. Journal of Medical Virology.

[ref11] (2020). Characteristics and outcomes of children with coronavirus disease 2019 (COVID-19) infection admitted to US and Canadian pediatric intensive care units. Journal of American Medical Association Pediatrics.

[ref12] (2020). Severe and fatal forms of COVID-19 in children. Archives of Pediatrics.

[ref13] (2020). Characteristic of COVID-19 infection in pediatric patients: early findings from two italian pediatric research networks. European Journal of Pediatrics.

[ref14] (2020). What should PRM specialists do? A clinician’s perspective. European Journal of Physical and Rehabilitation Medicine.

[ref15] (2020). Impact on rehabilitation programs during COVID-19 containment for children with pediatric and perinatal stroke. European Journal of Physical and Rehabilitation Medicine.

[ref16] (2009). Occupational therapy home programs for cerebral palsy: double-blind, randomized, controlled trial. Pediatrics.

[ref17] (2019). Botulinum toxin modulates posterior parietel cortex activation in post stroke spasticity of upper limb. Frontiers in Neurology.

[ref18] (2019). Botulinum toxin in the management of children with cerebral palsy. Pediatric Drugs.

[ref19] (2020). Information about the new coronavirus disease (COVID-19). Radiolagia Brasileira.

[ref20] (2017). Adverse drug reactions of botulinum neurotoxin type A in children with cerebral palsy: a pharmaco-epidemiological study in VigiBase. Developmental Medicine and Child Neurology.

[ref21] (2015). Questionnaire about the adverse events and side effects following botulinum toxin A treatment in patients with cerebral palsy.

[ref22] (2010). The updated european consensus 2009 on the use of botulinum toxin for children with cerebral palsy. European Journal of Paediatric Neurology.

[ref23] (2011). Methods of formal consensus in classification/diagnostic criteria and guideline development. Seminars in Arthritis and Rheumatism.

[ref24] (2003). Standardized reporting of clinical practice guidelines: a proposal from the conference on guideline standardization. Annals of Internal Medicine.

[ref25] (2017). Guidance on conducting and reporting delphi studies (CREDES) in palliative care: Recommendations based on a methodological systematic review. Palliative Medicine.

[ref26] (2018). The impact of patient, intervention, comparison,outcome as search strategy tool on literature search quality on literature search quality: a systematic review. Journal of the Medical Library Association.

[ref27] (2020). What we should know and what we should do. Seminars in Cardiothoracic and Vascular Anesthesia.

[ref28] (2010). Systemic adverse events following botulinum toxin A therapy in children with cerebral palsy. Developmental Medicine and Child Neurology.

[ref29] (2018). Systemic adverse events after botulinum neurotoxin A injections in children with cerebral palsy. Developmental Medicine and Child Neurology.

[ref30] (2013). Safety of botulinum toxin A in children and adolescents with cerebral palsy in a pragmatic setting. Toxins.

[ref31] (2020). Minimizing sars-cov-2 exposure when performing surgical interventions during the COVID-19 pandemic. Journal of Neurointerventional Surgery.

[ref32] (2020). Air, surface environmental, and personal protective equipment contamination by severe acute respiratory syndrome coronavirus 2 (SARS-CoV-2) from a symptomatic patient. Journal of American Medical Association.

[ref33] (2020). Practical guidance for outpatient spasticity management during the coronavirus (COVID-19) pandemic: canadian spasticity COVID-19 task force. Canadian Journal of Neurological Sciences.

[ref34] Russell, Hugo and Ayliffe’s Principles and Practice of Disinfection, Preservation and Sterilization. Blackwell. doi: 10.1002/9780470755884.

[ref35] (2020). Ultrasound-guided interventions during the COVID-19 pandemic - a new challenge. American Journal of Physical Medicine and Rehabilitation.

[ref36] (2020). Peri-operative and critical care concerns in coronavirus pandemic. Indian Journal of Anaesthesia.

[ref37] Peripheral nerve blocks in a patient with suspected COVID-19 infection. Journal of ClinicalAnesthesia2020; 65: 109853. doi: 10.1016/j.jclinane2020.109853.

[ref38] (2020). Perspectives for the use of therapeutic Botulinum toxin as a multifaceted candidate drug to attenuate covid-19. Drug Discovery.

[ref39] (2020). Coronavirus (the invisible killer). Libera Publishing.

[ref40] (2020). Detection of SARS-CoV-2 in different types of clinical specimens. Journal of American Medical Association.

[ref41] (2020). Sars-cov-2 infection in children. New England Journal of Medicine.

[ref42] (2020). ISBN : 978-605-136-477-3.

